# Resolution of organ functional scores to predict outcomes in severe acute respiratory distress syndrome patients receiving extracorporeal membrane oxygenation

**DOI:** 10.1186/cc10707

**Published:** 2012-03-20

**Authors:** L Chiu, Y Chen, H Hu, C Huang, F Tsai, K Kao

**Affiliations:** 1 Chang Gung Memorial Hospital, Taoyuan, Taiwan

## Introduction

Extracorporeal membrane oxygenation (ECMO) may be used as an alternative therapy for severe acute respiratory distress syndrome (ARDS) patients who have failed conventional mechanical ventilation. We undertook a study to investigate the determinants of mortality and the sequential evolution of organ failures in ECMO-treated ARDS patients.

## Methods

This was a prospective observational study of severe ARDS patients who received venovenous ECMO in the ICU of Chang Gung Memorial Hospital between March 2006 and December 2010. We included data on all 38 consecutive patients who receive venovenous ECMO. Retrospective data included the following: demographics, primary diagnosis for ARDS, ventilator setting before ECMO, oxygenation, durations of ECMO, SOFA scores and outcome.

## Results

A total of 38 severe ARDS patients receiving ECMO were eligible. The causes of ARDS in these 38 patients were pneumonia in 21 patients, trauma in 10 patients, sepsis in three patients, pulmonary hemorrhage in two patients and others in two patients. The overall hospital mortality rate was 39% (15/38). Compared with the nonsurvivors group, the survivors group was younger (33.3 ± 15.1 vs. 52.2 ± 18.1 years old, *P *= 0.001) and had lower APACHE II scores (18.7 ± 6.3 vs. 26.7 ± 6.6, *P *= 0.001) and SOFA scores (10.4 ± 2.7 vs. 12.7 ± 2.4, *P *= 0.014). Furthermore, the survivors group had early significant resolution in the sequential SOFA scores compared with the nonsurvivors group.

## Conclusion

Survivors had early improvements in SOFA scores after ECMO for severe ARDS patients. SOFA score evolution may be used for evaluating the effect of ECMO.

